# LCP1 promotes ovarian cancer cell resistance to olaparib by activating the JAK2/STAT3 signalling pathway

**DOI:** 10.1080/15384047.2024.2432117

**Published:** 2024-11-26

**Authors:** Minxue Gai, Lanlan Zhao, Hongqi Li, Guoyu Jin, Wei Li, Fei Wang, Ming Liu

**Affiliations:** a Medical Integration and Practice Center, Shandong University, Jinan, Shandong, China; b Department of Gynecology, Shandong Provincial Hospital, Shandong University, Jinan, Shandong, China; c Department of Gynecology, Shandong Traditional Chinese Medicine University, Jinan, Shandong, China; d Department of Obstetrics and Gynecology, Qilu Hospital of Shandong University, Jinan, Shandong, China

**Keywords:** PARPi_1_, resistance2, ovarian cancer3, LCP1_4_, JAK2/STAT3_5_, EMT_6_

## Abstract

**Background:**

Resistance to poly (ADP-ribose) polymerase (PARP) inhibitors (PARPis) remain a major challenge in ovarian cancer (OC) treatment. However, the underlying mechanism of PARPi resistance is still poorly characterized. Increasing evidence has proven that lymphocyte cytosolic protein 1 (LCP1) promotes tumor progression. The JAK2/STAT3 signaling pathway plays an important role in increasing tumor metastatic ability and chemoresistance in cancer by promoting epithelial – mesenchymal transition (EMT).

**Methods:**

We established an olaparib-resistant OC cell line and studied its toxicologic effects through cell survival, Transwell, colony formation, western blotting and flow cytometry assays. RNA sequencing and screening were then performed to identify genes associated with olaparib resistance. Lymphocyte cytosolic protein 1 (LCP1) was found to be overexpressed in olaparib-resistant OC cells.

**Results:**

The inhibition of cell survival and promotion of cell apoptosis induced by olaparib in parental cells were significantly attenuated in olaparib-resistant cells. LCP1 was upregulated in olaparib-resistant cells compared with parental OC cells. Moreover, we found that the protein levels of JAK2/STAT3 signaling pathway components and EMT markers were increased in olaparib-resistant cells. Overexpression of LCP1 increased olaparib resistance in OC cells, and knockdown of LCP1 attenuated olaparib resistance. The changes in the protein levels of JAK2/STAT3 signaling pathway members and EMT markers between the cell types were similar to the changes in the levels of LCP1.

**Conclusions:**

These findings indicate that LCP1 expression may play an important role in the resistance of OC to olaparib by activating the JAK2/STAT3 signaling pathway and EMT. LCP1 could be a potential therapeutic target for patients with OC who are resistant to olaparib. Our study provides a new mechanism of olaparib resistance.

## Introduction

Currently, ovarian cancer (OC) is attracting increasing attention worldwide because it is one of the most common causes of gynecologic cancer-related death in women. Approximately 200 000 women die of OC worldwide each year.^1^ Due to the many difficulties in the early detection of OC, more than 75% of patients are diagnosed at an advanced stage, and these patients have a 5-year overall survival rate that is lower than 30%.^2,3^ After first-line chemotherapy, PARP inhibitors (PARPis), such as olaparib, are approved for maintenance therapy in patients with advanced OC.^4^ With the clinical application of PARPis, 35% of patients develop resistance to PARPis.^5,6^ Despite extensive research, the mechanism underlying PARPi resistance in OC has not been elucidated. Therefore, exploring the exact mechanism underlying PARPi resistance in OC is urgently needed.

The actin-binding protein lymphocyte cytosolic protein 1 (LCP1) was initially shown to be beneficial for the immune response in hematopoietic cells.^7,8^ Guido. et al demonstrated that immune synapse stability was increased by activation and enrichment of immune synapses in naïve and effector T cells.^8^ Moreover, LCP1 was shown to be expressed in many nonhaematopoietic cancers, and it was shown to be correlated with lymph node metastasis in prostate cancer.^9^ Additionally, LCP1 overexpression induces the proliferation and metastasis of colorectal cancer^10^ and oral cancer^11^ cells. According to a report by Bao et al., LCP1 may play a crucial role in the resistance of gastric cancer to chidamide.^12^ However, there are currently few reports on the role of LCP1 in OC.

In this study, we aimed to investigate the potential mechanism underlying PARPi resistance. We chose SKOV3 and A2780 cells, which are both PARPi-sensitive cells, to study resistance to olaparib.^13^ We established an OC cell line with acquired olaparib resistance and then performed transcriptome analysis of olaparib-resistant OC cells and parental cells to identify the genes involved in olaparib resistance in OC. We found that LCP1 was overexpressed in olaparib-resistant cells.

## Materials and methods

### Reagents and antibodies

The PARP inhibitors that we used in our study included olaparib (MedChemExpress [MCE], USA), Pamiparib (Beijing Beigene Medicine Ltd., China) and fluzoparib (Jiangsu Hengrui Medicine Co., Ltd., China). The JAK2 inhibitor we used in this study was fedratinib (MCE, USA) at a working concentration of 2 μM. The primary antibodies that were used for western blotting in our study included anti-LCP1 (Proteintech, China), anti-cleaved PARP (Cell Signaling Technology [CST], USA), anti-caspase-3 (CST, USA), anti-N-cadherin (Abcam, UK), anti-E-cadherin (CST, USA), anti-vimentin (Santa Cruz Biotechnology, USA), anti-JAK2 (Santa Cruz Biotechnology, USA), anti-phospho-JAK2 (Abcam, UK), anti-STAT3 (Santa Cruz Biotechnology, USA), anti-phospho-STAT3 (CST, USA), anti-α-tubulin (Proteintech, China) and anti-glyceraldehyde 3-phosphate dehydrogenase (GAPDH; CST) antibodies. Horseradish peroxidase (HRP)-conjugated secondary antibodies (Proteintech, China) were used for western blotting.

## Cell lines and culture

We purchased human OC cell lines (SKOV3 and A2780 cells) from the Chinese Academy of Sciences. SKOV3 cells were maintained in RPMI 1640 medium (GIBCO, USA) supplemented with 10% fetal bovine serum (FBS) (GIBCO, USA) and 100 units/mL penicillin/streptomycin (P/S) (GIBCO, USA). A2780 cells were cultured in DMEM-H supplemented with high glucose (GIBCO, USA), 10% FBS (GIBCO, USA) and 100 units/mL penicillin/streptomycin (P/S) (GIBCO, USA). All the cells were incubated at 37°C in humidified air with 5% carbon dioxide (CO_2_). Mycoplasma contamination was monitored on a monthly basis in all the cultures.

## RNA‑Sequencing and bioinformatics analysis

SKOV3 cells and SKOV3R cells were cultured at a confluence of 70–80%. Total RNA was isolated with TRIzol reagent (Thermo Fisher Scientific) and then snap frozen in liquid nitrogen immediately. Libraries were constructed using a U-mRNAseq Library Prep Kit (AT4221, KAITAI-BIO) with a Ribo-off rRNA Depletion Kit (Bacteria) (N407 Vazyme). Libraries were pooled and sequenced using the Illumina NovaSeq machine as 150 bp paired-end sequencing reads.

## Cell counting kit-8 (CCK-8) viability assay

Cell viability was assessed by using a Cell Counting Kit-8 (CCK-8; MCE, USA). Cells were seeded in 96-well plates (5,000 cells per well) and cultured in DMEM-H or RPMI 1640 medium supplemented with 10% FBS and 1% PS. After 24 hours, different concentrations of the drugs were added. After 72 hours, 10 μL of CCK-8 reagent was added to each well, after which the cell viability was determined by measuring the OD at 450 nm with a microplate spectrophotometer.

## Generation of a PARPi-resistant cell line

Olaparib resistance was induced in tumor cells by culturing the cells in increasing concentrations of olaparib. The IC50 value of olaparib was determined via a CCK-8 assay, and the cells were cultured with 1/5 of the IC50 value (approximately 7 μM in SKOV3 cells) as the initial concentration. When cell growth significantly slowed or when only 30%-40% of the cells survived, the culture medium was discarded, the cells were washed twice with PBS, and the medium was replaced with drug-free medium. After the cells were restored, they were digested and passaged. This process was repeated until the cells continued to grow stably at this concentration of olaparib. The process lasted approximately 3–4 weeks. These steps were repeated with increasing concentrations of various drugs. The concentration of olaparib gradually increased from 7 μM to 150 μM until a stable number of cells survived after treatment with various concentrations of olaparib. The concentration was increased until the cells exhibited obvious drug resistance. When more than 90% of the cells survived in medium supplemented with 150 μM olaparib for 24 hours, the establishment of olaparib-resistant SKOV3 cells was considered successful. Drug resistance persisted after cryopreservation and resuscitation. The resistance index (RI) was calculated according to the following formula:

$$\mathrm{Resistancee}\;\mathrm{index}\;(\mathrm{RI})=\frac{\mathrm{IC}\;50\;\mathrm{Resistant}\;\mathrm{cell}}{\mathrm{IC}\;50\;\mathrm{Parent}\;\mathrm{cell}}$$

## Plasmid construction and transfection

Syngen-tech plasmids (Beijing, China) were used to up- or downregulate LCP1 expression in OC cells. Cells were transfected with PCMV-LCP1, PCMV, sh-LCP1①, sh-LCP1② or PLKO when they were 30–50% confluent. Stably transfected cell lines were selected by incubation with puromycin for 1 week. The transfection efficiency was examined by qRT‒PCR and western blotting analysis.

LCP1 shRNA①(human): pLV-hU6-LCP1 shRNA01 (human)-hef1a-mNeongreen-P2A-hluc-P2A-Puro

LCP1 shRNA② (human): pLV-hU6-LCP1 shRNA02 (human)-hef1a-mNeongreen-P2A-hluc-P2A-Puro

LCP1 shNC (human):pLV-hU6-NC shRNA03-hef1a-mNeongreen-P2A-hluc-P2A-Puro

CMV-LCP1: pLV-hef1a-mNeongreen-P2A-Puro-WPRE-CMV-LCP1 (NM_002298, human)-3flag

CMV: pLV-hef1a-mNeongreen-P2A-Puro-WPRE-CMV-MCS-3flag

LCP1 shRNA01 (human): GCAATGGATACATCAGCTTCA

LCP1 shRNA02 (human): GCCTTCAGCCTTAATGGAAGG

LCP1 shNC (human):AAACGTGACACGTTCGGAGAA

## Assessment of apoptosis with annexin V-APC/7-aad

After olaparib treatment for 72 hours, 1 × 10^6^ cells were washed with PBS and stained with Annexin V-APC (5 µL) and 7-AAD (5 µL) in 1× binding buffer (BioGems, USA) for 15 minutes in the dark. The stained cells were analyzed with a BD FACSCalibur flow cytometer and FlowJo software.

## Colony formation assay

Cells were plated in six-well plates (2000 cells/well). After olaparib treatment for 14 days, the colonies were fixed with 4% paraformaldehyde for 30 minutes and stained with 0.5% crystal violet for 30 minutes. The number of colonies was determined via ImageJ software.

## Wound healing assay

Cells were plated in six-well plates (6 × 105 cells/well). After 18 h, the cell monolayer was scraped using a pipette tip of 20ul, washed with PBS, and examined under the microscope. After 24 hours, the wound area was photographed under a microscope. The relative cell migration rate was calculated by ImageJ.

## Transwell assay

Migration and invasion were evaluated using 24-well Transwell plates with membrane inserts containing 8.0-μm pores (Corning, NY). After 24 h of starvation, the appropriate cells were added to the upper chambers in serum-free medium, and 700 µL of medium supplemented with 10% FBS was added to the lower chambers. After 18 hours (migration) or 36 hours (invasion) at 37°C, the cells in the upper chambers were removed with a cotton swab, and the cells that were attached to the bottom of the membrane were fixed with 4% paraformaldehyde and stained with 0.5% crystal violet. After air drying, the cells were imaged and counted with a microscope.

## Western blotting

The cells were washed with PBS and resuspended in Western Lysis Buffer (Beyotime) supplemented with 1 mm PMSF and 1 mm phosphatase inhibitor. After centrifugation at 6,000×g for 5 minutes, the cell supernatants were collected. The protein concentrations were measured with a BCA Protein Assay Kit (Beyotime). Thirty micrograms of protein was separated via SDS‒PAGE. After the proteins were fully separated, they were transferred to PVDF membranes. The membranes were incubated with specific primary antibodies and HRP-conjugated secondary antibodies. Protein expression was visualized with an enhanced ECL detection kit (Beyotime). The grayscale values of the bands were analyzed by ImageJ.

## qRT‒PCR

Total RNA was isolated with TRIzol reagent (Thermo Fisher Scientific). The mRNA was reverse transcribed using an RT reagent kit (Accurate Biology, China). Real-time PCR was performed using 10 μL of SYBR Premix Ex Taq (Accurate Biology, China) with a Fast Real-Time PCR System. The mRNA expression of the target genes was normalized to the mRNA expression of GAPDH using the 2−ΔΔCt method.

The primer sequences for LCP1 were as follows:

forward, 5′-GGGGTTCCTGGTCATACACC-3′,

reverse, 5′-CAAGCAAGCAGCCTTGAACA-3′.

The primer sequences for GAPDH were as follows:

forward, 5′- GGAGCGAGATCCCTCCAAAAT-3′,

reverse, 5′-GCCATCACGCCACAGTTTC-3′.

## Statistical analysis

All the values are reported as the mean ± the standard error of the mean (SEM). Statistical significance was assessed using the t test for comparisons between two groups. *p* < .05 was considered to indicate statistical significance.

## Results

### Establishment of an olaparib-resistant cell line

**Figure 1.**
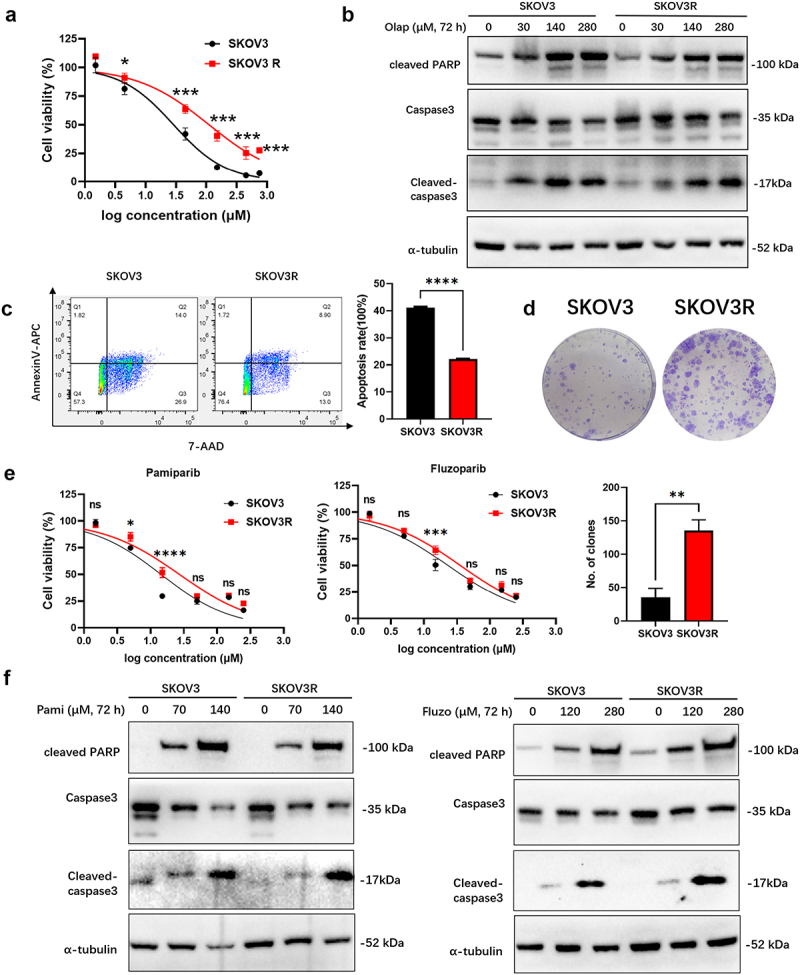
Characterization of the sensitivity of olaparib-resistant OC cells to olaparib. (a) The viability rate of SKOV3 and SKOV3R cells exposed to olaparib (0-750 μM) was assessed via a CCK-8 assay. (b) SKOV3 and SKOV3R cells were treated with 0 μM, 30 μM, 140 μM, or 280 μM olaparib for 72 hours. Then, the protein expression of cleaved PARP, caspase-3 and cleaved caspase-3 was measured. (c) SKOV3 and SKOV3R cells were treated with 30 μM olaparib for 72 h, and the apoptotic cells were stained with annexin V/7-AAD and analyzed by flow cytometry. (d) The colony formation ability of SKOV3 and SKOV3R cells treated with 5 μM olaparib for 2 weeks was analyzed. (e) The viability rate of SKOV3 and SKOV3R cells exposed to pamiparib (0-250 μM) and fluzoparib (0-250 μM) was assessed via a CCK-8 assay. (f) SKOV3 and SKOV3R cells were treated with 0 μM pamiparib or fluzoparib or with pamiparib or fluzoparib at concentrations 5-fold or 10-fold greater than the IC50 for 72 hours, after which the protein expression of cleaved PARP, caspase-3 and cleaved caspase-3 was measured. The data are shown as the mean±sem; *, *p* < .05, **, *p* < .01, ***, *p* < .001, ****, *p* < .0001.

SKOV3 ovarian tumor cells were made resistant to olaparib by treating the cells with a concentration gradient of olaparib. The concentration of olaparib was gradually increased from 7 μM to 150 μM until a stable number of cells survived after treatment with various concentrations of olaparib. A resistant cell line was successfully generated after 9 months, and the drug resistance remained stable after 20–30 passages (Fig. S1). The cells were named olaparib-resistant SKOV3 (SKOV3R) cells. The control SKOV3R cells were obtained from SKOV3 parent cells after 9 months of passage. The half maximal inhibitory concentration (IC50) of olaparib in SKOV3R cells (117.30 μM) was greater than that in SKOV3 cells (28.72 μM), and the resistance index (RI) was 4.1 (Figure 1a). PARP is one of the main cleavage targets of Caspase-3. The cleaved PARP protein is used as a marker of apoptosis.^14^ SKOV3 parental cells and SKOV3R cells were treated with 0 μM, 30 μM, 140 μM, or 280 μM olaparib for 72 hours, after which the expression of the cleaved PARP protein and caspase-3 protein were measured (Figure 1b). At the same concentration of olaparib, the expression of apoptotic proteins (cleaved PARP and cleaved caspase-3) in SKOV3R cells were significantly decreased. Furthermore, olaparib-induced apoptosis was significantly reduced in SKOV3R cells (Figure 1c). A colony formation assay showed that after treatment with the same concentration of olaparib, SKOV3R cells had better colony formation ability than did SKOV3 cells (Figure 1d). After we removed the drug for a period of time, the drug resistance of the SKOV3R cells did not change. The above results confirmed that we successfully constructed a SKOV3R cell line. We measured the viability of SKOV3 parental cells and SKOV3R cells treated with the new PARP inhibitors pamiparib and fluzoparib. The IC50 of pamiparib was greater in SKOV3R cells (27.09 μM) than in SKOV3 parental cells (14.34 μM), with an RI of 1.89. The IC50 of fluzoparib was greater in SKOV3R cells (35.43 μM) than in SKOV3 parental cells (23.09 μM), with an RI of 1.53 (Figure 1E). Moreover, SKOV3 parental cells and SKOV3R cells were treated with pamiparib and fluzoparib at 0 μM, 5-fold the IC50 and 10-fold the IC50 for 72 hours. Analysis of the expression level of the cleaved PARP and cleaved caspase-3 protein showed that SKOV3 parental cells and SKOV3R cells both underwent significant apoptosis after treatment with certain concentrations of pamiparib and fluzoparib (Figure 1F). In summary, we believe that SKOV3R cells are sensitive to the new PARP inhibitors pamiparib and fluzoparib.

## Biological characteristics of olaparib-resistant OC cells

**Figure 2.**
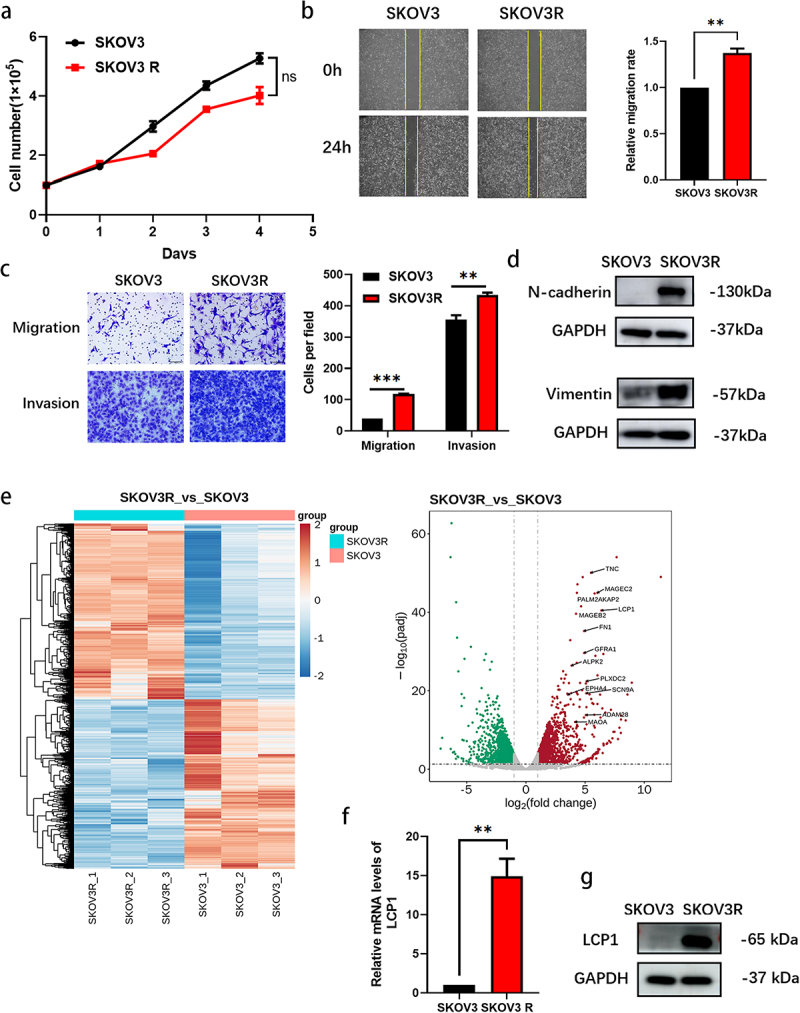
Biological characteristics and expression of LCP1 in olaparib-resistant OC cells. (a) A total of 1 × 10^5^ cells were seeded in a six-well plate, and the number of cells was counted after 1-5 days. (b) The migration abilities of SKOV3 and SKOV3R cells were detected via wound healing assay. (c)The migration and invasion abilities of SKOV3 and SKOV3R cells were detected via transwell assays. (d) The expression of EMT proteins in SKOV3 and SKOV3R cells was confirmed by western blot analysis. (e) Heatmap and (left) volcano plot (right) showing the genes that were differentially expressed between SKOV3 and SKOV3R cells. LCP1 was identified as one of the most highly upregulated genes in SKOV3R cells compared with SKOV3 cells. (f) qRT‒pcr was used to measure the mRNA levels of LCP1 in olaparib-resistant OC cells. (g) The expression of LCP1 in SKOV3 and SKOV3R cells was confirmed by western blot analysis.

The growth rate of SKOV3R cells was similar to that of the parental cells, and the difference was not statistically significant (Figure 2a). However, the migration and invasion abilities of SKOV3R cells were significantly greater than those of SKOV3 parental cells (Figure 2b,c). It has been suggested that epithelial ovarian cancer (EOC) cells may undergo an epithelial mesenchymal transition (EMT)-like process during localized invasion in the peritoneum and retain mesenchymal features in advanced tumors.^15^ This mainly occurs as a result of a reduction in the number of intercellular junctions resulting from reduced cell‒cell adhesion due to loss of E-cadherin function or expression and an increase in N-cadherin and vimentin expression. In fact, the expression of the mesenchymal markers N-cadherin and vimentin was greatly increased in SKOV3R cells (Figure 2d). We explored the protein expression of E-cadherin in SKOV3 and SKOV3R cells, but the western blot results were not consistent. We hypothesized that the expression of E-cadherin was too low to be detected or that there was a problem with the experimental method. These findings provide insight into the mechanism of tumor recurrence and metastasis in patients with olaparib-resistant ovarian cancer.

## LCP1 is overexpressed in olaparib-resistant ovarian cancer cells

To explore the molecular mechanisms of ovarian cancer cell resistance to olaparib, RNA sequencing of SKOV3 parental cells and SKOV3R cells was performed, after which differences in transcriptional profiles were analyzed. A total of 3961 genes were differentially expressed between SKOV3R cells and SKOV3 parental cells, with 2016 genes exhibiting upregulated expression and 1945 genes exhibiting downregulated expression (Figure 2e). Among these genes, one of the most upregulated genes, lymphocyte cytosolic protein 1 (LCP1), attracted our attention. LCP1 is normally expressed in hematopoietic cells during the immune response. Furthermore, increased expression of LCP1 has been linked to prostate cancer progression.^16^ LCP1 was shown to be a critical mediator of AP4-dependent prostate cancer progression.^17^ LCP1 could be an effective therapeutic target for patients with chidamide resistance. However, studies on its role in ovarian cancer, especially in patients with PARP inhibitor-resistant ovarian cancer, are rare.^18^ Therefore, LCP1 was selected for further study. qRT‒PCR and western blot results showed that the expression of LCP1 was upregulated in SKOV3R cells (Figure 2f, g).

## LCP1 drives olaparib resistance in ovarian cancer cells

**Figure 3.**
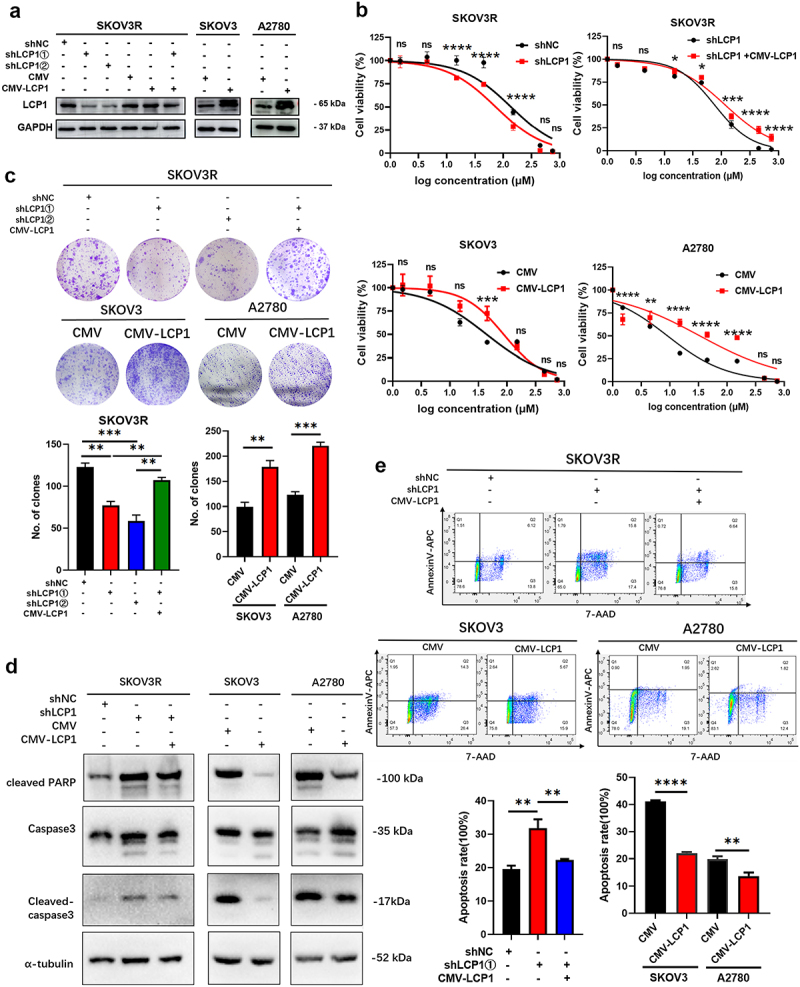
LCP1 drives olaparib resistance in OC cells. (a) LCP1 expression in LCP1-knockdown and LCP1-overexpressing OC cells was confirmed via western blotting. (b) After exposure to olaparib (0-750 μM), the viability of LCP1-knockdown and LCP1-overexpressing OC cells was examined by a CCK-8 assay. (c) Colony formation assays were performed with LCP1-knockdown or LCP1-overexpressing OC cells that were treated with 3 μM olaparib for 2 weeks. (d) LCP1-knockdown and LCP1-overexpressing OC cells were treated with olaparib for 72 hours, after which the protein expression of cleaved PARP, caspase-3 and cleaved caspase-3 was measured. (e) LCP1-knockdown and LCP1-overexpressing SKOV3 cells were treated with 30 μM olaparib for 72 h. LCP1-overexpressing A2780 cells were treated with 10 μM olaparib for 72 h. The apoptotic cells were stained with annexin V/7-AAD and analyzed by flow cytometry.

To determine whether LCP1 expression is involved in the resistance of ovarian cancer cells to olaparib, we constructed cell lines with LCP1 knockdown or overexpressing. The overexpression or knockdown efficiency of LCP1 in these cells was subsequently assessed via western blotting (Figure 3a). The IC50 of olaparib in A2780 cells (9.09 μM) was lower than that in SKOV3 cells (28.72 μM), which indicated that A2780 cells are more sensitive to olaparib (Figure 3b). The CCK-8 assay demonstrated that knockdown of LCP1 sensitizes SKOV3R cells to olaparib treatment. Consistently, overexpression of LCP1 increased the ovarian cancer cell survival rate in the presence of the same concentration of olaparib (Figure 3b). A colony formation assay showed that after treatment with the same concentration of olaparib, colony formation ability was significantly decreased in cells with LCP1 knockdown and increased in cells with LCP1 overexpression (Figure 3c). According to the western blot analysis of LCP1 expression, we selected shLCP1①-transfected SKOV3R cells, which exhibited improved LCP1 knockdown, for flow cytometry and western blot experiments. The protein level of cleaved PARP and cleaved caspase-3 increased after LCP1 knockdown but decreased after LCP1 overexpression (Figure 3d). Moreover, the results of flow cytometry showed that overexpression of LCP1 could reduce the apoptosis of ovarian cancer cells induced by olaparib (Figure 3e). Subsequently, we transfected CMV-LCP1 lentivirus into LCP1-knockdown SKOV3R cells. After selection with puromycin, we obtained new SKOV3R cell lines. The above experiments were repeated, and it was found that overexpressing LCP1 reversed the increase in drug sensitivity caused by LCP1 knockdown (Figure 3a, b, c, d, e). Thus, these results suggested that LCP1 mediates the sensitivity of ovarian cancer cells to olaparib.

## LCP1 promotes the metastasis of olaparib-resistant OC cells

**Figure 4.**
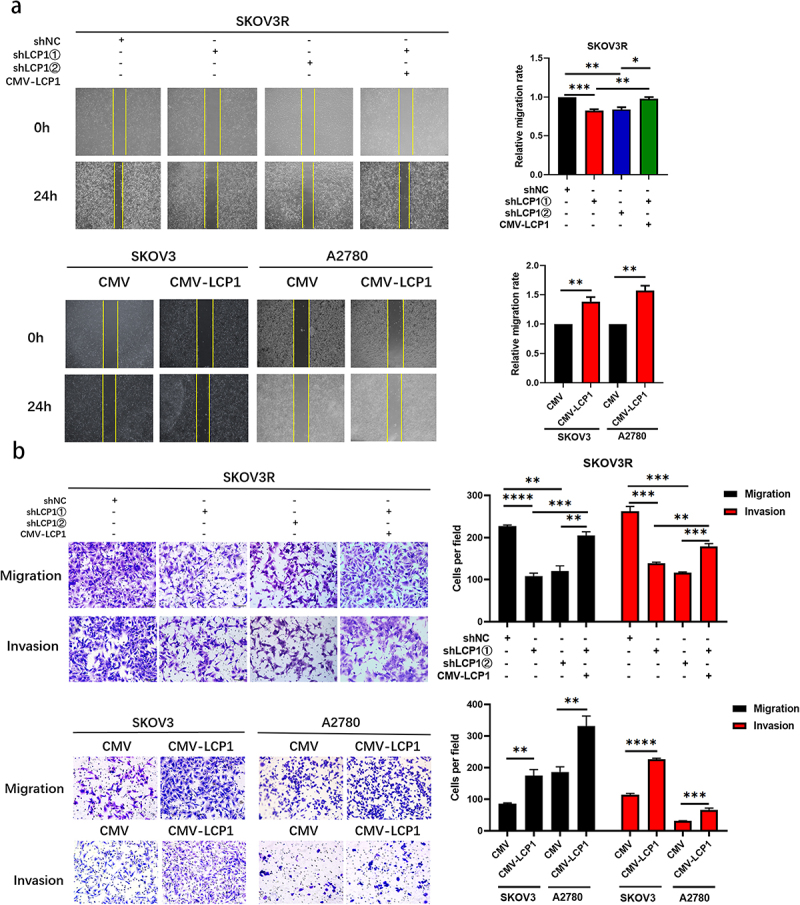
LCP1 promotes the abilities of migration and invasion in ovarian cancer cells(a) the migration abilities of LCP1-knockdown and LCP1-overexpressing OC cells were detected via wound healing assay. (b) The migration and invasion abilities of LCP1-knockdown and LCP1-overexpressing OC cells were detected via transwell assays.

**Figure 5.**
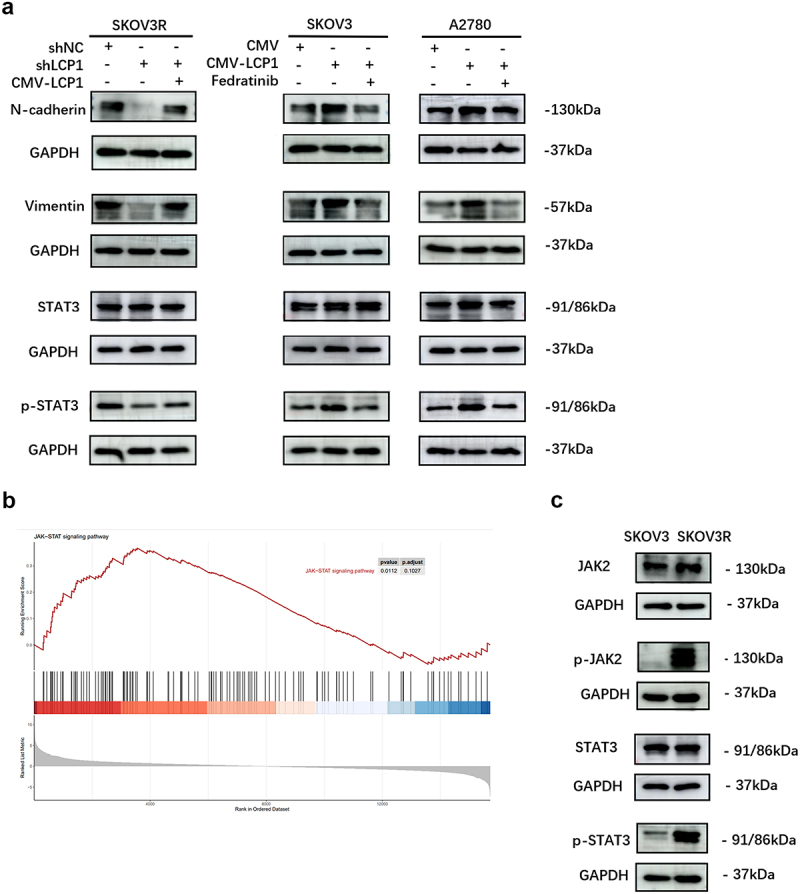
LCP1 promotes olaparib resistance by activating the JAK2/STAT3 signaling pathway and EMT. (a) The expression of STAT3 and EMT proteins in LCP1-knockdown and LCP1-overexpressing OC cells treated with fedratinib or not was confirmed by western blot analysis. (b) Single-gene GSEA showed that the JAK/STAT signaling pathway was enriched in SKOV3R cells. (c) The protein expression of JAK2/STAT3 pathway in SKOV3 and SKOV3R cells was confirmed by western blot analysis.

Ovarian cancer patients who take olaparib for maintenance therapy usually experience recurrence of OC accompanied by metastasis of ovarian cancer lesions. To verify whether LCP1 plays an important role in OC cell metastasis, we conducted wound healing assay and Transwell assay. Relative cell migration rate decreased significantly after LCP1 knockdown but increased after LCP1 overexpression in wound healing assay, and Transwell assay also showed similar changes (Figure 4a,b). Thus, the migration and invasion ability of SKOV3R cells were significantly decreased after LCP1 was knocked down. When LCP1 was overexpressed in cells with LCP1 knockdown, the opposite effects were observed. In contrast, compared with SKOV3 parental cells, LCP1-overexpressing OC cells showed increased migration and invasion abilities after olaparib treatment. We assessed the relationship between LCP1 and EMT-related proteins by western blotting. The expression of N-cadherin and vimentin was attenuated in LCP1-knockdown OC cells, and this effect was reversed by overexpressing LCP1. The opposite result was observed in the LCP1-overexpressing cells (Figure 5a). Based on these findings, we can conclude that LCP1 promotes the metastasis of ovarian cancer cells by activating EMT.

## LCP1 promotes olaparib resistance by activating the JAK2/STAT3 signaling pathway and EMT

To reveal the downstream signaling pathway of LCP1, genes in a predefined gene set were ranked according to the degree to which they were differentially expressed in SKOV3R cells compared with SKOV3 parental cells. That is, gene set enrichment analysis (GSEA) was performed. The results indicated that JAK/STAT signaling pathway activity was increased in SKOV3R cells (Figure 5b). The JAK2/STAT3 signaling pathway can promote EMT to enhance the migration and invasion abilities of ovarian cancer cells.^19^ It has been reported that the exosomal transfer of LCP1 promotes the tumorigenesis and metastasis of osteosarcoma cells by activating the JAK2/STAT3 signaling pathway.^20^ Therefore, we speculate that the mechanism of PARPi resistance in ovarian cancer cells may be related to enhancement of the activity of this pathway. Western blot assay indicated that the expression of p-JAK2 and p-STAT3 proteins was increased in SKOV3R cells (Figure 5c). And LCP1 knockdown markedly decreased the phosphorylation of JAK2 and STAT3 in SKOV3R cells. The opposite result was observed in the LCP1 overexpression group (Figure 5a). To explore the relationships between LCP1 and JAK2/STAT3 and EMT, LCP1-overexpressing OC cells were treated with the JAK2 inhibitor fedratinib (TG101348) for 12 hours. The results showed that fedratinib decreased the expression of the downstream protein phosphorylated STAT3 and mesenchymal markers N-cadherin and vimentin proteins in LCP1-overexpressing cells (Figure 5a). Thus, our results indicated that LCP1 can promote EMT by activating the JAK2/STAT3 signaling pathway to promote the resistance of ovarian cancer cells to olaparib.

## Discussion

The successful development of PARPis has provided an effective therapeutic option for ovarian cancer; this is a prime example of success in precision medicine. PARP inhibitors can cause acquired resistance via three general mechanisms. These mechanisms include the restoration of HR function, protection of the replication fork, and mutation of the DNA-binding domain of PARP.^21^ However, there is no universally accepted PARPi resistance mechanism. To overcome PARPi resistance and improve PARPi sensitivity, the optimal combination of PARPi therapy and other treatment options urgently needs to be determined.

In this study, we assessed the resistance of SKOV3 cells, which are classic cells in the study of ovarian cancer, to olaparib. The resistance of SKOV3 cells to olaparib has been reported in many articles. Although the IC50 of olaparib in SKOV3 cells varies among these studies, all studies have suggested that SKOV3 cells are sensitive to olaparib.^13,22,23^ Based on these findings, a SKOV3 cell line resistant to olaparib was constructed to explore the mechanism of olaparib resistance in OC cells. We reported that abnormal expression of LCP1 led to ovarian cancer cell resistance to olaparib by activating the JAK2/STAT3 signaling pathway and EMT. LCP1 is overexpressed in olaparib-resistant ovarian cancer cells, and downregulation of LCP1 can increase olaparib-induced apoptotic cell death and the sensitivity of ovarian cancer to olaparib. Our study showed that LCP1 is an attractive target for PARPi resistance diagnosis and therapy.

The actin-binding protein LCP1 is usually expressed in hematopoietic cells and participates in the immune response of hematopoietic cells.^24^ However, the LCP1 protein is often ectopically expressed in nonhematopoietic malignant cancer cells and is considered a marker of cancer progression.^9,25–27^ In particular, LCP1 phosphorylation at Ser5 has been shown to be important for the immune response of leukocytes and the invasion and migration of cancer cells.^27^ In this study, we found for the first time that LCP1 is overexpressed in olaparib-resistant ovarian cancer cells. We demonstrated that LCP1 can promote EMT by activating the JAK2/STAT3 signaling pathway in ovarian cancer cells, thereby promoting tumor cell metastasis and invasion. The LCP1/JAK2/STAT3 axis is a key signaling pathway involved in olaparib resistance in ovarian cancer. In the present study, we detected several EMT-related proteins after treatment with JAK2 inhibitors to determine whether LCP1 promotes EMT by activating the JAK2/STAT3 signaling pathway. However, additional cell characteristics should be examined in combination with the use of JAK/STAT inhibitors in our future studies. Ovarian cancer patients who are resistant to olaparib rarely undergo resurgery; thus, accessing relevant tissues for clinical experiments is very difficult. This is a major limitation of the present study. First, we overexpressed LCP1 in olaparib-resistant ovarian cancer cells and then conducted relevant experiments to explore its relationship with olaparib resistance. However, there are currently no studies exploring the impact of LCP1 on the development of ovarian cancer cells. The focus of our next study will be to conduct relevant in vivo and in vitro experiments to explore the impact of changing LCP1 expression on ovarian cancer. In addition, conducting relevant experiments with tissues from ovarian cancer patients who underwent surgery to explore whether high LCP1 expression is associated with poor survival or increased risk of recurrence is crucial for future studies.

In summary, in this study, we demonstrated that LCP1 is involved in olaparib resistance in ovarian cancer cells by activating the JAK2/STAT3 pathway and EMT. Our study provides a new mechanism of PARPi resistance and multiple therapies for overcoming PARPi resistance.

## Availability of data and materials

The raw RNA sequencing data used in this study are available in the Sequence Read Archive (SRA) database under accession number PRJNA1030959.

## Supplementary Material

figureS1.tif

## List of abbreviations

poly (ADP-ribose) polymerase inhibitor: PARPi
ovarian cancer: OC
lymphocyte cytosolic protein 1: LCP1
epithelial – mesenchymal transition: EMT
the half maximal inhibitory concentration: IC50
resistance index: RI
epithelial ovarian cancer: EOC
gene set enrichment analysis: GSEA
