# Supplementary figures and images for: LCP1 promotes ovarian cancer cell resistance to olaparib by activating the JAK2/STAT3 signalling pathway
Source: Cancer Biol Ther. 2024 Nov 26;25(1):2432117. doi: 10.1080/15384047.2024.2432117 (PMC11601053; doi:10.1080/15384047.2024.2432117)

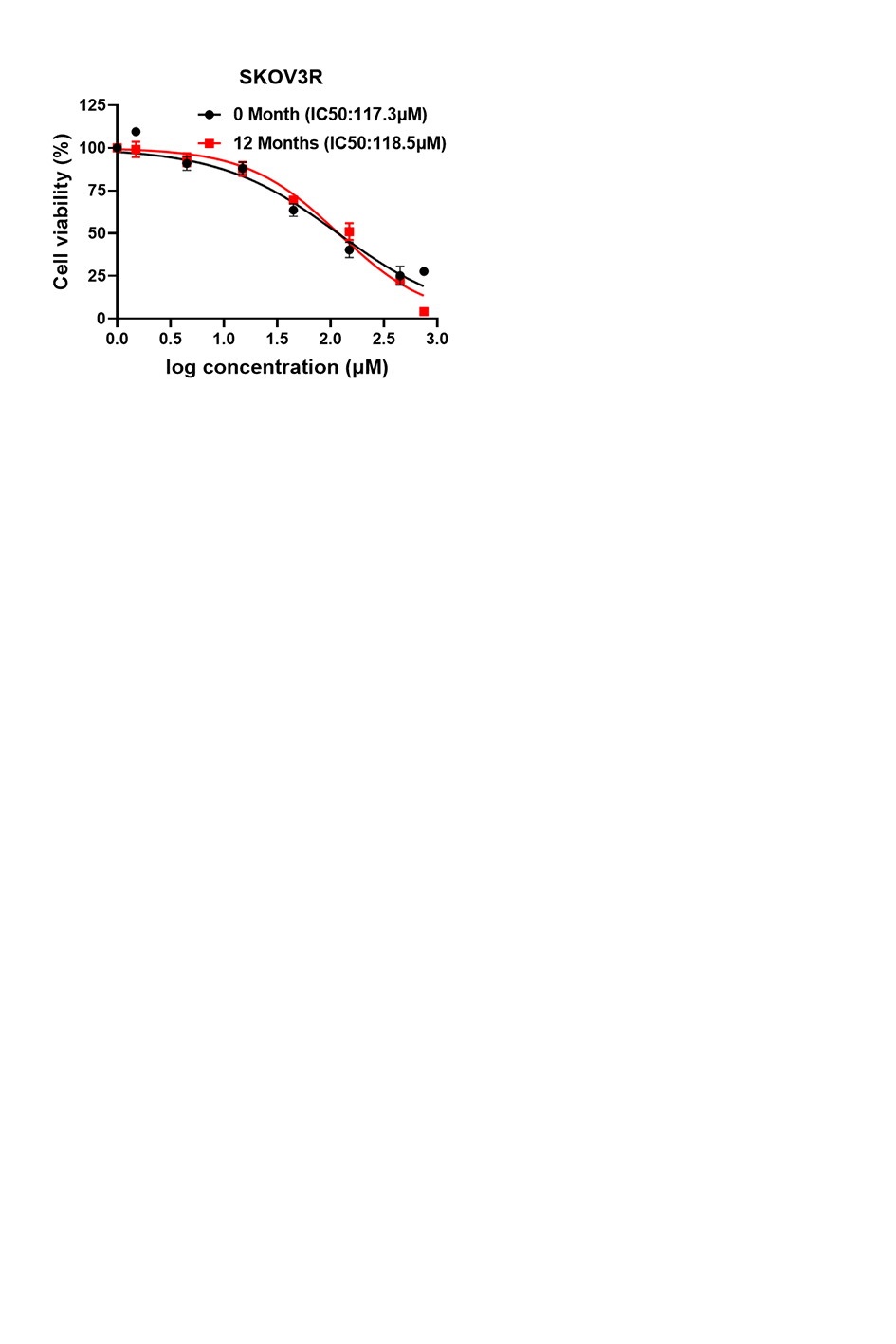

Supplement: figureS1.tif [file KCBT_A_2432117_SM3604.tif]
